# Changes in Clinical Profile, Treatment, and Mortality in Patients Hospitalised for Acute Myocardial Infarction between 1985 and 2008

**DOI:** 10.1371/journal.pone.0026917

**Published:** 2011-11-02

**Authors:** Sjoerd T. Nauta, Jaap W. Deckers, Martijn Akkerhuis, Mattie Lenzen, Maarten L. Simoons, Ron T. van Domburg

**Affiliations:** Department of Cardiology, Erasmus Medical Center, Rotterdam, The Netherlands; University of Modena and Reggio Emilia, Italy

## Abstract

**Objectives:**

To quantify the impact of the implementation of treatment modalities into clinical practice since 1985, on outcome of patients with ST-segment elevation myocardial infarction (STEMI) or non-ST-segment elevation myocardial infarction (NSTEMI).

**Methods:**

All consecutive patients admitted for STEMI or NSTEMI at the Thoraxcenter between 1985 and 2008 were included. Baseline characteristics, pharmacological and invasive treatment modalities, and survival status were collected. The study population was categorised in three groups of patients: those hospitalised between 1985–1990, 1990–2000, and 2000–2008.

**Results:**

We identified 14,434 patients hospitalised for myocardial infarction (MI). Both STEMI and NSTEMI patients were increasingly treated with the current guideline based therapy. In STEMI, at 30 days following admission, cumulative mortality rate decreased from 17% in 1985–1990 to 13% in 1990–2000, and to 6% in 2000–2008. Adjusted 30-day and three-year mortality in the last period was 80% and 68% lower than in 1985, respectively. In NSTEMI, at 30 days following admission, cumulative mortality rate decreased from 6% in 1985–1990 to 4% in 1990–2000, and to 2% in 2000–2008. Adjusted 30-day and three-year mortality in the last period was 78% and 49% lower than in 1985, respectively. For patients admitted between 2000 and 2008, 3 year survival of STEMI and NSTEMI patients was 87% and 88%, respectively.

**Conclusions:**

Our results indicate substantial improvements in acute- and long-term survival in patients hospitalised for MI, related to improved acute- as well as long-term treatment. Early medical evaluation in suspected MI and intensive early hospital treatment both remain warranted in the future.

## Introduction

During the last 25 years, the management of patients presenting with an acute myocardial infarction (MI) has undergone many transformations. Until 1984, treatment was limited to providing symptomatic relief, and management of complications as arrhythmia's, acute heart failure, or post-infarction angina. In the 1980s, the introduction of antithrombotic treatment with aspirin and intravenous (or intracoronary) fibrinolysis resulted in significant mortality reductions in patients with ST-segment elevation myocardial infarction (STEMI).[1] In the nineties, pre-hospital identification (“triage”) of patients with an acute myocardial infarction with an indication for reperfusion therapy and subsequent immediate (pre-hospital) initiation of thrombolytic treatment was introduced in some areas.[2], [3] Although more effective thrombolytic agents became available,[4] reperfusion of the infarct-related vessel often failed,[5] and bleeding complications were a limiting factor of fibrinolyis.[6] Gradually, mechanical percutaneous techniques improved, and in the last decade primary percutaneous coronary intervention (PCI) became the treatment of choice in patients presenting with a STEMI.[7], [8]

In the same time period, patients with non-ST-elevation myocardial infarction (NSTEMI) benefitted from improved anti-thrombotic and anti-coagulant therapy,[9] better risk stratification and tailored treatment with selective coronary revascularization in high risk patients.[9], [10], [11], [12] Additionally, effective secondary prevention was introduced with aspirin, beta-blockers, statins, and ACE inhibitors in subjects with LV dysfunction and, subsequently, in high risk MI survivors.[13], [14], [15], [16], [17] In combination, all these developments reshaped the treatment map of the patient with an MI.[18], [19], [20]

The impact of the implementation of all these treatment modalities into clinical practice on outcome has not yet been fully quantified. Therefore, we analysed changes in clinical practise, treatment, and 30-day as well as three-year outcome in a consecutive series of STEMI or NSTEMI patients admitted at our institution, an academic tertiary referral center, between 1985 and 2008.

## Methods

We included all consecutive patients aged >18 years admitted for STEMI or NSTEMI to the Intensive Coronary Care Unit (ICCU) of the Thoraxcenter, Erasmus University Medical Center between June 1985 and December 2008. The Thoraxcenter was the referral center for all PCIs in the Rotterdam region until 2005 when a second hospital started a PCI programme. Regional arrangements were made such that patients with MI were referred to either hospital according to a pre-arranged schedule.

The primary discharge diagnosis of MI was made in the presence of the following characteristics: chest pain or equivalent symptoms in combination with dynamic ECG changes consistent with MI and a serial rise (to at least three times the upper normal value) and fall in serum biochemical markers of cardiac necrosis such as creatine kinase-MB and troponin T (as of 2002). Patients were diagnosed as STEMI in the presence of ST-segment elevation > 0.1 mV in at least two peripheral leads, or > 0.2 mV in at least two precordial leads, and as NSTEMI otherwise. For patients admitted more than once, only the first hospitalisation was taken into account.

### Data collection

This is a prospective observational study. Trained physicians and nurses accustomed to the use of standardized case report forms collected the data. Demographic characteristics (age, gender), cardiac history (previous MI, PCI or coronary artery bypass surgery [CABG]), risk factors (hypertension, diabetes, family history, smoking status), renal dysfunction (creatinine value >150 µmol/L), and pharmacological and invasive treatment modalities (thrombolysis and PCI) were collected.

### Follow-up and endpoints

The primary endpoint was all cause mortality at 30 days and at three-years. In-hospital mortality was retrieved from the medical records. Survival status was assessed through municipal Civil Registries in 2010 and was available for 99% of all patients.

### Ethics Statement

This study has been approved by the Ethical Committee of the Erasmus Medical Center, Rotterdam. According to Dutch laws, informed consent is not required for register-based research of pre-existing personal data. Therefore, the Ethical Committee waived the need for informed consent.

### Statistical Analysis

Data are summarized as frequencies and percentages for categorical variables. Continuous variables are presented as mean and standard deviation or median and 25th and 75th percentile. The study population was categorised in three groups of patients, those hospitalised between 1985–1990; 1990–2000; and 2000–2008, respectively. These categories were chosen according to important improvements in therapy with complete introduction of thrombolysis in 1991 and substantial increase in the use of primary PCI in 2001. Categorized variables among the three groups were compared with the chi-square test and continuous variables by ANOVA with Bonferroni corrections. In addition, we compared changes in mortality in time periods of three year, and these changes are presented in Forrest plots.

Cumulative survival and one-minus-survival curves were constructed using the Kaplan–Meier method according to date of hospitalization as presented above. A log-rank test was used to compare survival curves. We examined the independent association between year of hospitalization and mortality using logistic regression for 30-day outcome and the Cox proportional hazards model for long-term outcome, with adjustment for age, gender, previous MI, previous CABG, diabetes, hypertension and smoking status. Results are reported as odds or hazard ratios of mortality and their respective 95% confidence intervals. Proportionality of hazards was tested graphically by inspection of log–log survival curves and by a formal test of proportionality based on Schoenfeld residuals for each variable in the model. Calibration refers to whether the model agrees with the observed probabilities and was assessed with the Hosmer–Lemeshow statistic.

All statistical tests were 2-tailed, and p-values were significant at <0.05. Analysis was performed using SPSS software version 17.0 (SPSS, Chicago, USA).

## Results

We identified 14,434 consecutive patients hospitalised for MI in our center between 1985 and 2008: 6,820 STEMI and 7,614 NSTEMI patients. At three years follow-up, mortality had occurred in 2,190 patients, 1,232 in STEMI and 958 in NSTEMI patients.

### ST-segment elevation myocardial infarction

The characteristics of the STEMI patients are depicted in Table 1. With time, STEMI patients presented older, were more likely to have diabetes, hyperlipidemia, anemia, and a history of PCI. Further, STEMI patients were less likely to present with renal dysfunction or a history of MI.

**Table 1:** Baseline characteristics, clinical presentation, and discharge medication of patients hospitalised for STEMI

	*Period of admission*			P
	1985-1990	1990-2000	2000-2008	
No. of patients	947	1928	3945	
*Baseline*				
Age (years)	60±11.6	60±12.7	61±12.6	0.01
Gender (male)	731 (77%)	1432 (74%)	2937 (75%)	0.20
*Cardiac history*				
Previous MI	334 (35%)	463 (24%)	1075 (27%)	<0.001
Previous PCI	43 (5%)	127 (7%)	653 (17%)	<0.001
Previous CABG	83 (9%)	124 (6%)	282 (7%)	0.09
*Risk factors*				
Hypertension	320 (34%)	549 (29%)	1295 (33%)	<0.01
Diabetes	79 (8%)	204 (11%)	571 (14%)	<0.001
Hyperlipidemia	76 (8%)	295 (15%)	919 (23%)	<0.001
Family history	201 (21%)	400 (21%)	1062 (27%)	<0.001
Current smoker	403 (43%)	675 (35%)	1518 (39%)	<0.001
Renal dysfunction	104 (11%)	229 (12%)	179 (5%)	<0.001
Anemia	309 (33%)	818 (42%)	1464 (37%)	<0.001
*Medication at ICCU discharge*				
Statin	0 (.)	NA	2996 (76%)	<0.001
Aspirin	97 (10%)	1200 (62%)	3510 (89%)	<0.001
Beta-blocker	412 (44%)	958 (50%)	2341 (59%)	<0.001
Calcium antagonist	306 (32%)	315 (16%)	174 (4%)	<0.001
Nitrates	220 (23%)	241 (13%)	190 (5%)	<0.001
Diuretics	334 (35%)	306 (16%)	321 (8%)	<0.001
ACE inhibitor or ARB	0 (.)	403 (21%)	1308 (33%)	<0.001

ACE, Angiotensin-Converting Enzyme; ARB, Angiotensin receptor blocker; CABG, coronary artery bypass grafting surgery; ICCU, intensive coronary care unit; MI, myocardial infarction; NA, not available; PCI, percutaneous coronary intervention; STEMI, ST-elevation MI.

**Figure 1:**
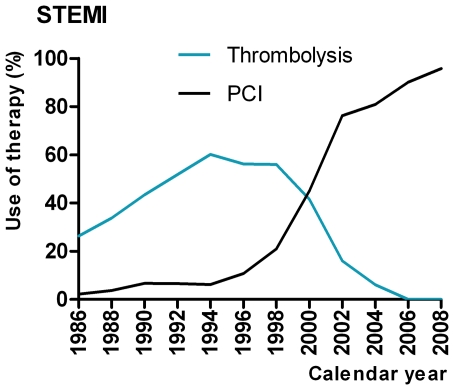
Treatment of STEMI patients over time

The percentage of STEMI patients treated with PCI increased gradually. Before 1985, neither intracoronary- nor intravenous thrombolytic therapy was employed, while intravenous fibrinolysis was the main treatment modality from 1985 to 1998. From 1998 onwards, treatment with thrombolysis was gradually replaced by primary PCI (Figure 1). Since our institution was initially the only hospital with PCI facilities in the region, the number of patients with STEMI admitted increased as a result of the decision to offer primary PCI to all STEMI patients in the Rotterdam Region. In the most recent cohort, drug therapy with aspirin, beta-blockers, statins, and ACE inhibitors or angiotensin receptor blockers was quite common, but calcium antagonists, diuretics or nitrates were prescribed less frequently.

**Figure 2:**
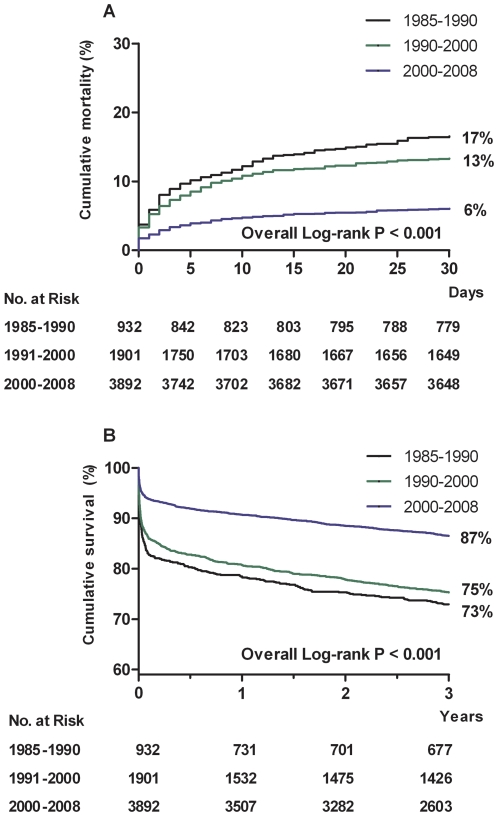
Kaplan-Meier curves for 30-day cumulative mortality (Figure 2A) or three-year cumulative survival (Figure 2B) according to calendar period of admission

**Figure 3:**
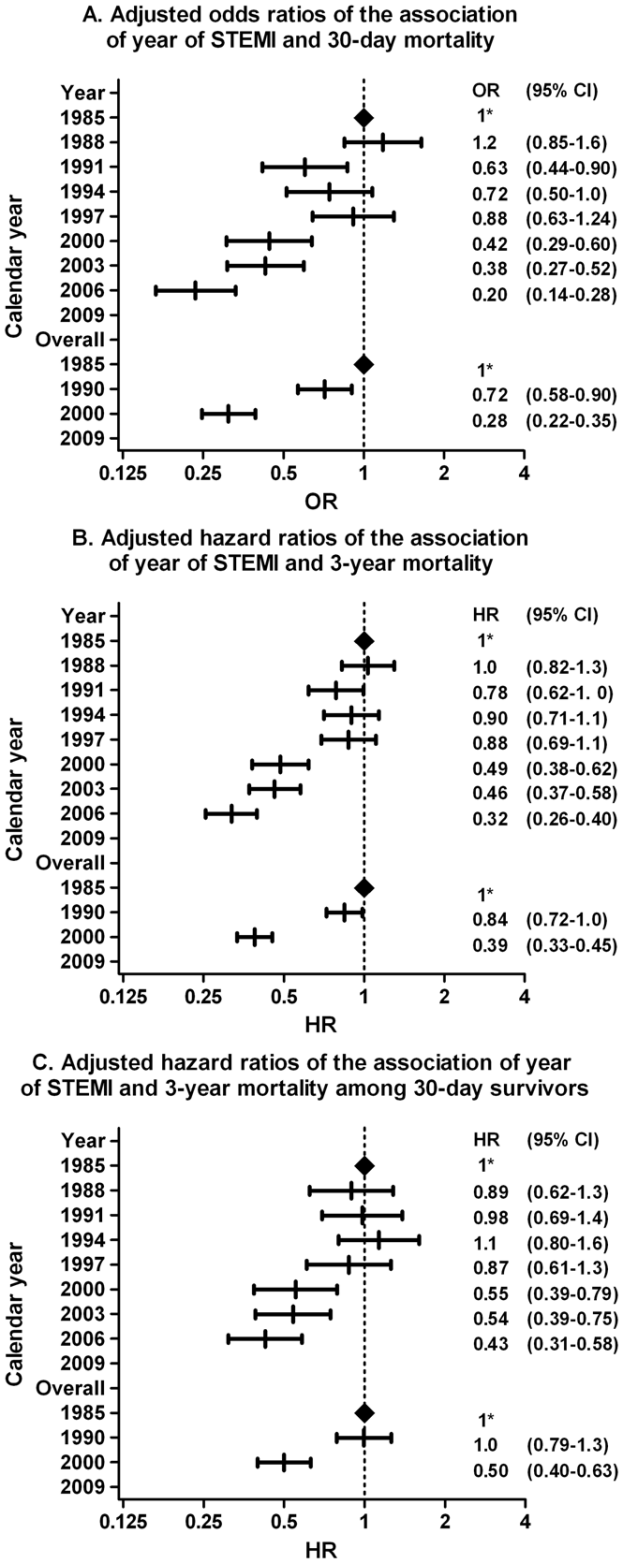
Adjusted odds or hazard ratios of secular trends in mortality after STEMI at 30-days (Figure 3A), at 3-year (Figure 3B), and at 3-year among 30-day survivors (Figure 3C). The upper part of the figure compares changes in mortality in time periods of three year, the lower part among patients hospitalised between 1985–1990; 1990–2000; and 2000–2008 *Reference category for the subsequent ratios CI, confidence interval; HR, Hazard ratio; OR, Odds ratio, STEMI, ST-elevation myocardial infarction

At 30 days following admission, cumulative mortality rate decreased from 17% in 1985-1990 to 13% in 1990-2000, and to 6% in 2000-2008 (p<0.001; Figure 2). Adjusted 30-day mortality in the last period was 80% lower than in 1985 (adjusted odds ratio [OR] 0.20, 95%CI 0.14-0.28, p<0.001) (Figure 3).

Cumulative three-year survival rate increased from 73% in 1985-1990 to 75% in 1990–2000 and to 87% in 2000–2008 (p<0.001). Adjusted three-year mortality in the last period was 68% lower than in 1985 (adjusted hazard ratio [HR] 0.32, 95%CI 0.26–0.40, p<0.001). In a landmark analysis, including only patients who survived the first 30 days, adjusted three-year mortality was 57% lower in the most recent period than in 1985 (adjusted HR 0.43, 95%CI 0.31-0.58, p<0.001, Figure 3).

### Non-ST-segment elevation myocardial infarction

With time, NSTEMI patients presented older, were more likely to have hypertension, diabetes, hyperlipidemia, or anemia and less likely to be a smoker (Table 2). Furthermore, NSTEMI patients more often presented with a history of PCI and less often presented with a history of MI in the most recent study period. As in STEMI, prescription of aspirin, beta-blockers, statins, and ACE inhibitors or angiotensin receptor blockers increased while the use of calcium antagonists and nitrates decreased.

**Table 2:** Baseline characteristics, clinical presentation, and discharge medication of patients hospitalised for NSTEMI

	*Period of admission*			P
	1985-1990	1990-2000	2000-2008	
No. of patients	1269	2672	3673	
*Baseline*				
Age (years)	61±10.3	63±11.7	63±11.8	<0.001
Gender (male)	921 (73%)	1806 (68%)	2575 (70%)	<0.01
*Cardiac history*				
Previous MI	581 (46%)	1050 (39%)	1290 (35%)	<0.001
Previous PCI	130 (10%)	356 (14%)	757 (21%)	<0.001
Previous CABG	196 (15%)	292 (12%)	390 (11%)	<0.001
*Risk factors*				
Hypertension	455 (36%)	881 (34%)	1604 (44%)	<0.001
Diabetes	95 (8%)	321 (12%)	745 (20%)	<0.001
Hyperlipidemia	159 (13%)	736 (24%)	1785 (49%)	<0.001
Family history	331 (26%)	666 (25%)	1122 (31%)	<0.001
Current smoker	459 (36%)	741 (28%)	880 (24%)	<0.001
Renal dysfunction	102 (8%)	222 (8%)	349 (10%)	0.20
Anemia	489 (39%)	1153 (43%)	1783 (49%)	<0.001
*Medication at ICCU discharge*				
Statin	0 (.)	NA	1962 (68%)	<0.001
Aspirin	225 (18%)	1169 (55%)	3125 (85%)	<0.001
Beta-blocker	865 (68%)	1271 (60%)	2334 (64%)	<0.001
Calcium antagonist	884 (70%)	972 (46%)	515 (14%)	<0.001
Nitrates	475 (37%)	393 (19%)	335 (9%)	<0.001
Diuretics	240 (19%)	276 (13%)	337 (9%)	<0.001
ACE inhibitor or ARB	0 (.)	191 (7%)	1134 (31%)	<0.001

ACE, Angiotensin-Converting Enzyme; ARB, Angiotensin receptor blocker; CABG, coronary artery bypass grafting surgery; ICCU, intensive coronary care unit; MI, myocardial infarction; NA, not available; NSTEMI, non-ST-elevation MI; PCI, percutaneous coronary intervention.

**Figure 4:**
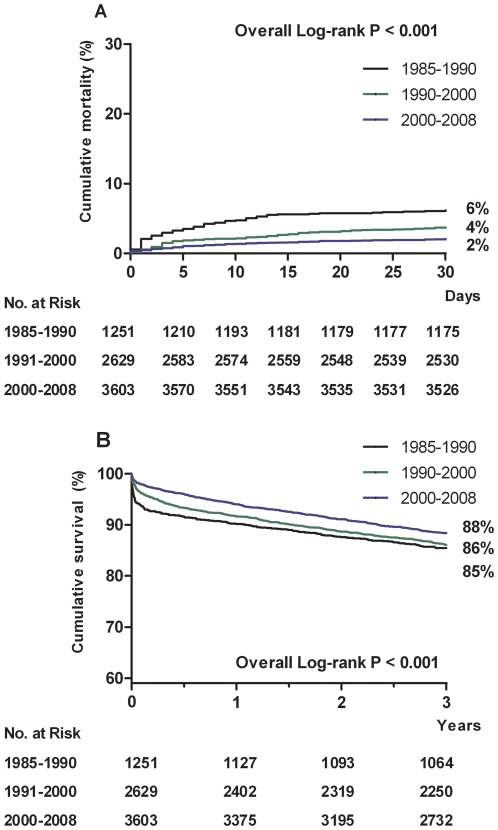
Kaplan-Meier curves for 30-day cumulative mortality or (Figure 4A) or three-year cumulative survival (Figure 4B) according to calendar period of admission

**Figure 5:**
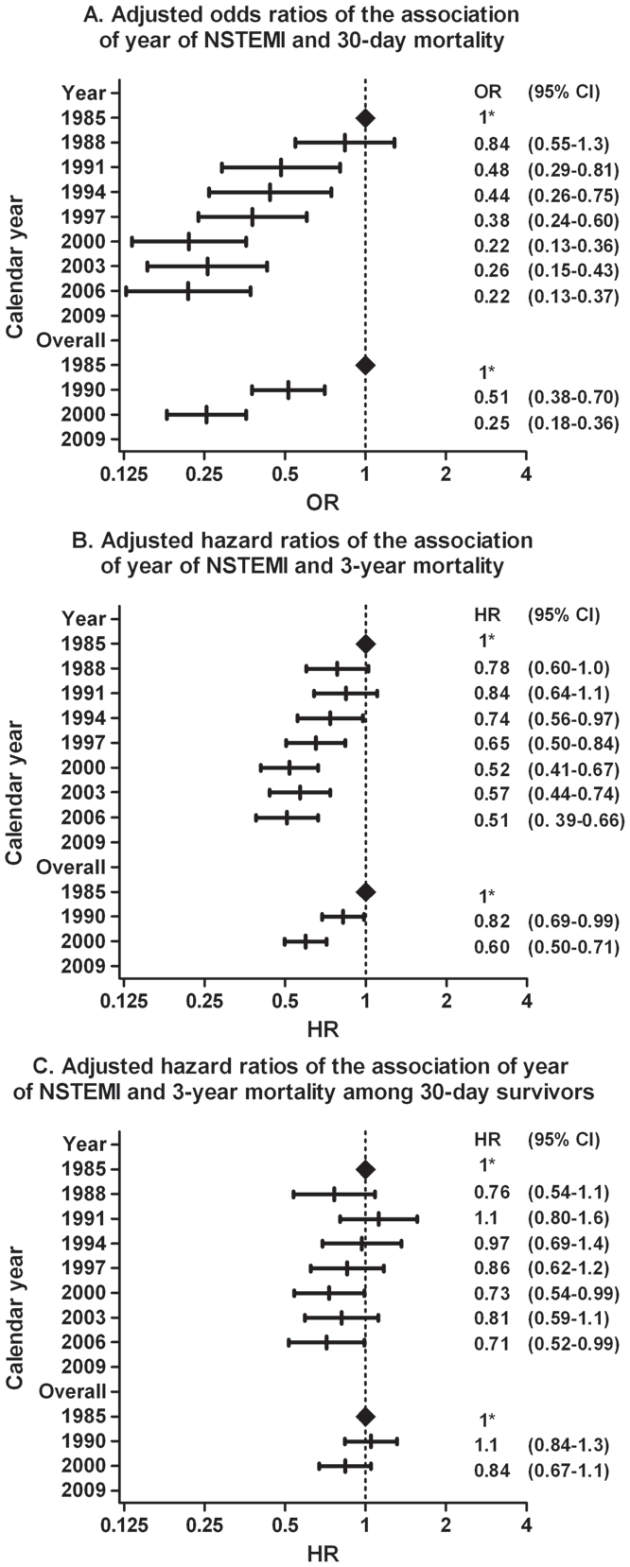
Adjusted odds or hazard ratios of secular trends in mortality after NSTEMI at 30- days (Figure 5A), at 3-year (Figure 5B), and at 3-year among 30-day survivors (Figure 5C). The upper part of the figures compares changes in mortality in time periods of three year, the lower part among patients hospitalised between 1985–1990; 1990–2000; and 2000–2008 *Reference category for the subsequent ratios CI, confidence interval; NSTEMI, non-ST-elevation myocardial infarction; HR, Hazard ratio; OR, Odds ratio

At 30 days following admission, cumulative mortality rate decreased from 6% in 1985-1990 to 4% in 1990–2000, and to 2% in 2000–2008 (p<0.001; Figure 4). Adjusted 30-day mortality in the last period was 78% lower than in 1985 (adjusted OR 0.22, 95%CI 0.13–0.37, p<0.001; Figure 5).

Cumulative three-year survival rate did not significantly change between 1985-1990 (85%) and 1990-2000 (86%). Three-year survival in the last period was 88% (p<0.001). Adjusted three-year mortality in the last period was 49% lower than in 1985 (adjusted HR 0.51, 95%CI 0.39-0.68, p<0.001). In a landmark analysis, including only patients who survived the first 30 days, adjusted three-year mortality was 29% lower in the most recent period than in 1985 (adjusted HR 0.71, 95%CI 0.52-0.99, p = 0.042, Figure 5).

For patients admitted between 2000 and 2008, long term survival of STEMI and NSTEMI patients was comparable, 87% and 88%, respectively (Figures 2 and 4). The higher subsequent mortality in non-STEMI patients resulted from their older age. After correction for this confounder, the risk of 30-day to three-year mortality was similar in NSTEMI and STEMI (adjusted HR 1.0, 95%CI 0.86-1.2).

## Discussion

We have shown that, during a period of almost 25 years, overall 30 day mortality in patients hospitalised for MI was reduced with 80%. In patients admitted for ST-segment elevation MI (STEMI), 30-day mortality declined from 17% to 6%. In patients with a smaller, non ST-segment elevation MI (NSTEMI) 30-day mortality declined from 6% to 2%. In addition, we demonstrated that this early survival benefit was maintained over three years of follow-up, with long term survival increasing from 73% to 87% and from 85% to 88% in STEMI and NSTEMI, respectively. The reduction in long- term all-cause mortality is illustrative of the strength of the improvement of the combined treatment effects.

There can be little doubt that such improvements have resulted from changes in medical therapy, both pharmacological as well as interventional.[20] The most important factors must have been the introduction of reperfusion therapy in the form of fibrinolytic therapy and later primary PCI for STEMI,[1], [8] as well as the more frequent use of revascularization in selected patients with NSTEMI. Also, the introduction and subsequent employment of medical therapies including statins, and ACE inhibitors or angiotensin receptor blockers must have played a role. Of course, patient characteristics also gradually changed during the observation period. However, most of the observed alterations in our patient's phenotypes, like increasing age and more co-morbidity such as diabetes and renal dysfunction, have previously been associated with impaired outcomes and do not account for the observed outcome improvement. A reduction in case fatality in spite of treating older patients with co-morbidity has also been observed in other observational studies.[20], [21], [22] Earlier clinical presentation could have contributed to improved prognosis, but we found no evidence for this.

Better primary prevention could have influenced the severity of the acute clinical event, and better secondary prevention must also have contributed significantly. Exact details of the medication use of our patients after discharge are not known, but registries in our region in 1995, 2001 and 2006 indicated that the use of preventive medication significantly intensified during that time.[14] For instance, statins were used by 36%, 72% and 93% of the patients respectively, with a subsequent drop in mean cholesterol level from 6.2 mmol/l in 1995 to 4.2 mmol/l in 2006. Aspirin was used by >90% of the patients from 1995 onwards, and smoking trends decreased from 32% to 15% between 1995 and 2006.[14]

With time, the number of patients admitted with myocardial infarction increased. This was mainly the result of changes in referrals: when primary PCI became the treatment of choice in STEMI patients, they were specifically referred to our center from 2000 onwards. Our STEMI patients can therefore be considered to be representative for this syndrome and we thus believe that the decrease in mortality in this group is broadly illustrative of current clinical practice. The number of NSTEMI patients also increased. Most likely, this was also due to changes in referrals, in particular of high risk patients in whom invasive therapy was warranted. Therefore, our population with this type of acute coronary syndrome will probably be less representative for that group at large. Still, given their already low mortality, the further decline in 30-day mortality in this group with time was also impressive.

Other studies, such as The National Registry of Myocardial Infarction, Worcester Heart Attack Study and Minnesota Heart Survey,[19], [20], [23], [24], [25], [26], [27], [28] have described prognostic time trends after acute MI, but none of these covered both i) secular trends for a period longer than 10 years from 1990 and ii) follow-up data after the first 30-days; as the present study does.

Although the present study thus has unique strengths, some of its limitations must be emphasized. First, the present data are derived from a single center. Although this will have enhanced the quality of the data and the observed secular trends, this could result in a lower external validity. However, this is unlikely, since a nation wide study in Sweden and a state wide study in the US reported quite comparable results.[21], [29] Second, based on our data, it is not possible to establish changes in MI incidence as other studies have shown. Still, the increasing age of our population provides indirect evidence of a lower incidence rate of myocardial infarction. Lastly, the current report is based on hospitalised patients, and we are therefore unable to assess the changes in mortality of all patients who suffered a myocardial infarction but died before clinical presentation.

### Conclusions

Our results indicate very substantial improvements in acute and long term survival in patients hospitalised for MI, related to improved acute- as well as long-term treatment. Since the early survival benefit is maintained over time, the present results emphasize that efforts to further reduce in-hospital mortality must be rigorously pursued, and that early medical evaluation in suspected MI and intensive early hospital treatment remain warranted. Although one might envisage even lower hospital mortality rates associated with acute MI in the future, absolute treatment benefits will become smaller because of the current low mortality levels. Indirectly therefore, our data emphasize the need for better and more effective primary prevention, as well as the necessity to target high risk MI subgroups such as the elderly and those with heart failure.
